# Editorial: Immunology and immunopathogenesis of human leishmaniasis

**DOI:** 10.3389/fcimb.2022.1055221

**Published:** 2022-10-14

**Authors:** Juliana P. B. de Menezes, Cláudia Brodskyn, Ricardo Gonçalves, Olivia Bacellar

**Affiliations:** ^1^ Laboratory of Parasite-Host Interaction and Epidemiology, Goncalo Moniz Institute, Salvador, Brazil; ^2^ General Pathology Department, Instituto de Ciências Biológicas (ICB), Federal University of Minas Gerais, Belo Horizonte, Minas Gerais, Brazil; ^3^ Servico de Imunologia, Complexo Hospitalar Universitario Prof. Edgard Santos, Universidade Federal da Bahia, Salvador, Brazil; ^4^ Instituto Nacional de Ciencia e Tecnologia de Doencas Tropicais - INCT-DT Conselho Nacional de Pesquisa/Ministério de Ciências e Tecnologia (CNPq/MCT), Salvador, Brazil

**Keywords:** human leishmaniasis, immunopathogenesis, IL-32, IL-17, peroxisome proliferator-activated receptor-γ (PPAR-γ), pioglitazone

Leishmaniasis is a disease that affects people and animals worldwide and can be broadly divided into visceral (VL) and tegumentary (TL) forms. TL caused by *Leishmania braziliensis* presents a wide spectrum de clinical manifestations ranging from a single and typical ulcer known as CL to the involvement of nasal or oral mucosal, mucosal leishmaniasis (ML). Between both clinical forms, there is diffuse cutaneous leishmaniasis (DCL) caused by *Leishmania amazonensis*. This spectrum included atypical cutaneous leishmaniasis (ACL) characterized by vegetative, verrucous, crusted and lupoid lesions (Guimarães et al., 2016). Human VL is characterized by depressed cell-mediated immunity and decreased Th1 immune response (Carvalho et al., 1989; Bacellar et al., 2000). Also, VL is associated with increased production of multiple pro-inflammatory cytokines and chemokines (Saporito et al., 2013; Palacios et al., 2021; Tasew et al., 2021).

Human CL caused by *Leishmania braziliensis* is characterized by an exacerbated cellular immune response and scarce numbers of parasites in the lesions (Bittencourt and Barral, 1991; Saldanha et al., 2017). The presence of pro-inflammatory cytokines, such as IFN-Y and TNF, are essential for the control of parasite proliferation, but total elimination of *Leishmania* does not occur, and an exaggerated Th1 immune response has been associated with severe inflammation and pathology (Ribeiro-de-Jesus et al., 1998; Bacellar et al., 2002; Antonelli et al., 2005; Carvalho et al., 2007; Novais et al., 2015; Santos et al., 2018; Amorim et al., 2019; Carvalho et al., 2020). The pentavalent antimony is the first-line drug for leishmaniasis treatment. However, an elevated rate of therapeutic failure rates has been reported, reaching 70% depending on the clinical form (Machado et al., 2002; Unger et al., 2009; Costa et al., 2018; Lago et al., 2018).

The aim of this Research Topic, “*Immunology and immunopathogenesis of human leishmaniasis*”, including five original research articles, is to deepen insight into the mechanisms of the innate and adaptive immune responses that are associated with the pathogenesis of human VL and CL as this may identify novel strategies that could be used to treat the disease. The main findings are briefly discussed in this editorial.

The pathogenesis of VL is complex, and the factors that cause the disease are not well understood. In this context, many changes in the gene expression profile may occur during infection development, including microRNAs (miRNAs). Ramos-Sanchez et al quantified changes in miRNAs associated with immune and inflammatory pathways using the *L*.*(L.) infantum* promastigote infected- human monocytic THP-1 cell model and plasma from patients with visceral leishmaniasis. They found that miR-548d-3p was one of the few miRNAs commonly regulated in THP-1 cells and plasma of VL patients. Additionally, transfection of THP-1 cells with a miR-548d-3p inhibitor revealed an effect on inhibiting *Leishmania* growth, likely through induction of MCP-1/CCL2 and nitric oxide production. These results extend previous observations of the author on the role of miR-548d-3p in cutaneous leishmaniasis caused by *L.braziliensis* (Souza et al., 2021). The miR-548d-3p may be explored as a biomarker of prognostic value, and targeting miR-548d-3p may provide new therapeutic opportunities in leishmaniasis.

Esteves et al., using a quantitative proteomics approach, identified 4,048 proteins, of which 254 and 196 showed increased and decreased abundance, respectively, in atypical cutaneous leishmaniasis strains compared to typical strains. Strains isolated from atypical forms showed an upregulation of proteins that influence the early stages of infection of the mammalian host and may favor parasite survival inside macrophages and proteins associated with resistance to antimony therapy.

Lesions of patients with ATL present a higher expression of IL-32γ. *L. amazonensis* and *L. braziliensis* induce this cytokine in human macrophages (Gomes et al., 2017) favoring the parasite’s control (dos Santos et al., 2017). Lipophosphoglycan (LPG) is a major multivirulence factor expressed on the *Leishmania* promastigote surface, and this molecule is essential during host-parasite interaction (de Assis et al., 2012). Silveira et al. investigated the ability of LPGs from *L.amazonensis* and *L. braziliensis* to induce IL-32 production in human peripheral blood mononuclear cells (PBMCs). They found that both LPGs induced IL-32γ, and this production was associated with production of IL-1β and IL-6 and was dependent of TLR4 and NOD. The authors suggest that the identification of parasite factors and host receptors responsible for IL-32γ production are important to the control of *Leishmania* and the development of new therapeutic strategies for treatment of this disease.

Th17 cells may play an essential role in protecting against certain extracellular and intracellular pathogens and have been demonstrated to influence the balance between inflammatory and anti-inflammatory cytokines (25). IL-17 is mainly produced by Th17 cells, and the effects of Th17/IL-17 are still unclear as disease-promoting and protective responses in the context of the different clinical forms of leishmaniasis (Anderson et al., 2009; Gonçalves-de-Albuquerque et al., 2017; Tomiotto-Pellissier et al., 2018). To understand the role of transcription factors (TFs) and genes involved in Th17 induction in *Leishmania*–macrophage interaction, Gonçalves et al. used gene regulatory networks (GRNs). The authors evaluated the dynamics of the infection profile correlated with the modulation of Th17 immune response in cutaneous leishmaniasis through data from transcriptome sequencing of macrophages infected with *L. major* and *L. amazonensis* associated with data experimentally validated in the literature. Their results showed that genes involved in the Th17 pathway are overexpressed on macrophages infected with both *Leishmania* species. At the initial stages, they observed the expression of genes related to immunological regulation; among these, genes that encode transcription factors STAT2, IL-2, and CXCL12 were identified as overexpressed.

Lastly, an increase in the therapy failure to antimony has been documented in CL patients, and several reports have shown that adjuvant therapy with anti-inflammatory drugs is beneficial to CL patients (Lessa et al., 2001; Machado et al., 2007; Carvalho et al., 2020). Nascimento et al. investigated the contribution of peroxisome proliferator-activated receptor-γ (PPAR-γ) activation by pioglitazone (an oral drug used in the treatment of diabetes) in the regulation of the inflammatory response and *L. braziliensis* killing by monocytes. They found that PPAR-γ activation by pioglitazone decreases the inflammatory response in CL patients without affecting *L. braziliensis* killing by monocytes, suggesting that this drug may serve as an adjunctive treatment for CL caused by *L. braziliensis*.

Collectively, the articles from this Research Topic presented important aspects of the role of inflammatory response in the pathogenesis of human leishmaniasis and the promising use of anti-inflammatory drugs in treating this disease.

## Author contributions

OB wrote the first draft and JM edited and commented on the draft. All authors contributed to the article and approved the submitted version

## Funding

This work was supported by the National Institutes of Health (AI 136032).

## Conflict of interest

The authors declare that the research was conducted in the absence of any commercial or financial relationships that could be construed as a potential conflict of interest.

## Publisher’s note

All claims expressed in this article are solely those of the authors and do not necessarily represent those of their affiliated organizations, or those of the publisher, the editors and the reviewers. Any product that may be evaluated in this article, or claim that may be made by its manufacturer, is not guaranteed or endorsed by the publisher.
